# IRF2–INPP4B-mediated autophagy suppresses apoptosis in acute myeloid leukemia cells

**DOI:** 10.1186/s40659-019-0218-7

**Published:** 2019-03-15

**Authors:** Feng Zhang, Jiajia Li, Junfeng Zhu, Lin Liu, Kai Zhu, Shuang Cheng, RuDi Lv, Pingping Zhang

**Affiliations:** 1 Department of Hematology, The First Affiliated Hospital of Bengbu Medical College, No. 287 Changhuai Road, Bengbu, 233004 Anhui People’s Republic of China; 2 Department of Hematology, Bengbu Medical College, No. 287 Changhuai Road, Bengbu, 233004 Anhui People’s Republic of China; 3 Department of Electrocardiogram, The First Affiliated Hospital of Bengbu Medical College, No. 287 Changhuai Road, Bengbu, 233004 Anhui People’s Republic of China

**Keywords:** IRF2, INPP4B, Autophagy, Apoptosis, Acute myeloid leukemia

## Abstract

**Background:**

The present study aimed to investigate the underlying role of interferon-regulatory factor 2 (IRF2)–inositol polyphosphate-4-phosphatase, type-II (INPP4B) axis in the regulation of autophagy in acute myeloid leukemia (AML) cells.

**Methods:**

Quantitative real time PCR (QRT-PCR) and western blot were performed to determine the expression levels of IRF2, INPP4B and autophagy-related markers in AML cell lines. Autophagy was assessed by elevated Beclin-1 expression, the conversion of light chain 3 (LC3)-I to LC3-II, downregulated p62 expression and green fluorescent protein (GFP)-LC3 puncta formation. The colony formation and apoptosis assays were performed to determine the effects of IRF2 and INPP4B on the growth of AML cells.

**Results:**

IRF2 and INPP4B were highly expressed in AML cell lines, and were positively correlated with autophagy-related proteins. Overexpression of IRF2 or INPP4B stimulated autophagy of AML cells, whereas inhibition of IRF2 or INPP4B resulted in the attenuation of autophagy. More importantly, IRF2 or INPP4B overexpression reversed autophagy inhibitor, 3-methyladenine (3-MA)-induced proliferation-inhibitory and pro-apoptotic effects, while IRF2 or INPP4B silencing overturned the proliferation-promoting and anti-apoptotic effects of autophagy activator rapamycin.

**Conclusion:**

IRF2–INPP4B signaling axis attenuated apoptosis through induction of autophagy in AML cells.

## Background

Acute myeloid leukemia (AML) is a hematopoietic malignancy characterized by the abnormal proliferation of undifferentiated myeloid precursors and impaired hematopoiesis [1]. As the most common type of acute leukemia in adults, AML develops rapidly, resulting in a low long-term survival rate, and its incidence increases with increasing age. Although 50–75% patients with AML have a response to chemotherapy, relapse represents the major cause of treatment failure [2]. So far, the pathogenesis of AML has not been fully elucidated.

It has become increasingly clear that autophagy might be involved in a variety of multiple biological processes (e.g., cell survival, aging and death) and implicated in metabolic diseases, tumors, degenerative diseases, aging and infection [3, 4]. Several lines of evidence suggest that autophagy and apoptosis share a signaling-dependent regulated process that allows the degradation of some cellular proteins in autophagosomes essential for maintaining cell homeostasis and organelle renewal [5]. At present, autophagy is generally regarded as a regulatory mechanism of defense and a survival response to stress [6]. Apoptosis, known as a form of programmed cell death, is a critical component in discarding unsalvageable cells or inhibiting overgrowth. Autophagy, on the other hand, initially attempts to save the injured cells. However, autophagy behaves oppositely and cooperates with apoptosis following metabolic stress subsequently accelerates cell death [7, 8]. Therefore, the imbalance between autophagy and apoptosis potentially leads to tumorigenesis. Liu and colleagues reported that autophagy-related gene 5 (Atg5)-dependent autophagy contributed to AML development [9]. Watson et al. demonstrated that loss of Atg5 resulted in an identical hematopoietic stem and progenitor population (HSPC) phenotype as loss of Atg7, confirming a general role for autophagy in HSPC regulation [10]. Moreover, Folkerts et al. showed that knockdown of Atg5 inhibited myeloid leukemia maintenance [11], indicating that targeting autophagy might provide new therapeutic options for treatment of AML.

The interferon regulatory factor (IRF) proteins family are the crucial factors in immunoregulation, cell proliferation regulation, hematopoietic stem cell development, lymphocyte differentiation and cellular response that is involved in tumorigenesis [12]. The IRF2 gene, a member of IRF family, is located on chromosome 4. Our previous study [13] demonstrated that shRNA-mediated IRF2 knockdown suppressed cell growth and colony formation, down-regulated the level of anti-apoptotic factor Bcl-2 and up-regulated the protein levels of apoptotic proteins Bax and the cleaved caspase 3 in AML cell lines OCI/AML-2, OCI/AML-3, and THP-1 cells. Further investigation showed that IRF2 upregulated inositol polyphosphate-4-phosphatase, type-II (INPP4B) expression via binding to INPP4B promoter, which in turn inhibited cell apoptosis in AML cells. Nevertheless, the detailed mechanism by which INPP4B inhibited AML cell apoptosis remained unclear. As was mentioned above, we hypothesis that IRF2 might regulate cell autophagy through interacting with INPP4B, thereby affecting the growth and apoptosis of AML cells, and ultimately participating in the induction of AML development.

## Materials and methods

### Cell lines

AML cell lines (OCI/AML-2, OCI/AML-3, Kasumi-3, PL-21, MV-4-11, CESS, Kasumi-1, BDCM and THP-1) purchased from American Type Culture Collection (ATCC, Manassas, VA, USA) were maintained in α-minimal essential medium (MEM) supplemented with 10% fetal bovine serum (FBS), 100 U/mL penicillin and 100 µg/mL streptomycin (all from Invitrogen, Carlsbad, CA, USA) at 37 °C in humidified 5% CO_2_ and 95% air.

### Transient transfections and treatments

Full-length IRF2 and INPP4B were amplified and cloned into the pcDNA3.1 expression vector which was then stably transfected into OCI/AML-2 or THP-1 cells for IRF2 and INPP4B overexpression, while small interference RNA (siRNA) targeting IRF2 (si-IRF2) and INPP4B (si-INPP4B) oligos and control siRNA (si-Ctrl) were used to construct the knockdown models and negative controls, which were all designed and synthesized by Shanghai GenePharma Co., Ltd. (Shanghai, China). The qRT-PCR and western blotting were used to detect the transfection efficiency. Additionally, the protein levels of autophagy-related genes Beclin-1, microtubule-associated protein light chain 3 (LC3)-I, LC3-II and p62 were also examined. The green fluorescent protein (GFP)-LC3 puncta formation was also evaluated by immunofluorescence.

OCI/AML-2 and THP-1 cells were transfected with IRF2, INPP4B, si-IRF2, si-INPP4B or their negative controls for 48 h using Lipofectamine 2000 (Invitrogen) after treatment with autophagy inhibitor 3-methyladenine (3-MA; 10 mmol/L; Sigma-Aldrich, St. Louis, MO, USA), autophagy activator rapamycin (10 μg/L; LC Laboratories, Woburn, MA, USA) or 0.1% dimethylsulfoxide (DMSO; Sigma-Aldrich) for another 24 h. Cells were collected for determining the expression of autophagy-related proteins, autophagosome accumulation, proliferation and apoptosis.

### Immunofluorescence

OCI/AML-2 or THP-1 cells following transfection with IRF2, INPP4B, si-IRF2, si-INPP4B or their negative controls were seeded into a 35 mm diameter petri dish covered with a glass slide (microscope cover glass, 18 × 18 mm) at a density of 8 × 10^4^ cells/mL and maintained at 37 °C with 5% CO_2_ for 24 h. These cells were transiently transfected with GFP-LC3 plasmid using Lipofectamine 2000 (Invitrogen) according to the manufacturer’s specification. After 48 h of posttransfection, the slides were rinsed with PBS for three times, fixed in 4% paraformaldehyde for 10 min, washed again with PBS for three times, and sealed with anti-fluorescence quenching mounting medium (15 μL). Fluorescence levels of GFP-LC3 were detected by using laser confocal microscopy (CLSM; Leica, Wetzlar, Germany; magnification 60×). To quantify autophagic cells, we counted the number of autophagic cells demonstrating GFP-LC3 dots (≥ 10 dots/cell) among 200 GFP-positive cells.

### RNA extraction and PCR analysis

Total RNA was extracted from AML cell lines using Trizol reagent (Invitrogen), and reversely transcribed into cDNA using the SuperScript III kit (Invitrogen) according to the manufacturer’s protocol. The relative quantification of IRF2, INPP4B, Beclin-1 and LC3 mRNA expression levels were determined using Real-Time Quantitative PCR SYBR Green kit (Takara, Tokyo, Japan) on an ABI 7500 Real-Time PCR system (Applied Biosystems, Carlsbad, CA, USA) and calculated by the 2^−ΔΔCt^ method. GAPDH served as an internal control.

### Western blot

Total protein was separated from AML cell lines using Radio-Immunoprecipitation Assay (RIPA) buffer (Santa Cruz Biotechnology, Santa Cruz, CA, USA), incubated with 6% sodium dodecyl sulfate polyacrylamide gel electrophoresis (SDS-PAGE; Beyotime, Shanghai, China) and transferred to polyvinylidene fluoride (PVDF) membranes (Bio-Rad, Hercules, CA, USA). Afterwards, the membranes were blocked with tris buffered saline tween (TBST; 1.5 mM Tris, 5 mM NaCl, 0.1% Tween20) containing 5% skim milk at room temperature for 1 h and probed with primary antibodies against IRF2, INPP4B, Beclin-1, LC3-I, LC3-II and p62 (1:1000 dilution; all from Cell Signaling Technology, Boston, MA, USA) at 4 °C overnight. The secondary antibody was horseradish peroxidase-labeled antibody (1:5000), and β-actin was used as an internal control. Band intensities were standardized and the relative density was analyzed on a Molecular Imager ChemiDoc XRS System (Bio-Rad Laboratories, Hercules, CA, USA) using enhanced chemiluminescence reagent (Thermo Scientific, Shanghai, China).

### Cell proliferation assay

The proliferation of OCI/AML-2 and THP-1 cells was assessed by colony formation assay. Approximately 5000 cells were seeded in 35 mm plates and incubated at 37 °C for 9 days. Subsequently, these cells were washed twice with PBS, and stained with 0.1% crystal violet (Beyotime, Shanghai, China) for 15 min at room temperature. The number of colonies was counted under an inverted microscope (Leica).

### Cell apoptosis assay

Cell apoptosis was analyzed by flow cytometry using Annexin V-FITC apoptosis detection kit (BD Biosciences; San Jose, CA, USA) according to the manufacturer’s protocols. OCI/AML-2 and THP-1 cells following transfection or different treatments were collected, washed with cold PBS and stained with binding buffer containing Annexin V-FITC and propidium iodide (PI) at 4 °C under darkness for 15 min. Finally, cells were recorded using flow cytometry (Beckman Coulter, Fullerton, CA, USA).

### Statistical analysis

Data were presented as mean ± standard deviation (SD) and analyzed using SPSS 22.0 (IBM, Armonk, NY, USA). Two or more data sets were compared using Student’s t-test or one-way analysis of variance, with P < 0.05 being considered statistically significant. Correlation analysis was performed using the Spearman’s rank test.

## Results

### IRF2 and INPP4B were positively correlated with autophagy-related genes in AML cells

**Fig. 1:**
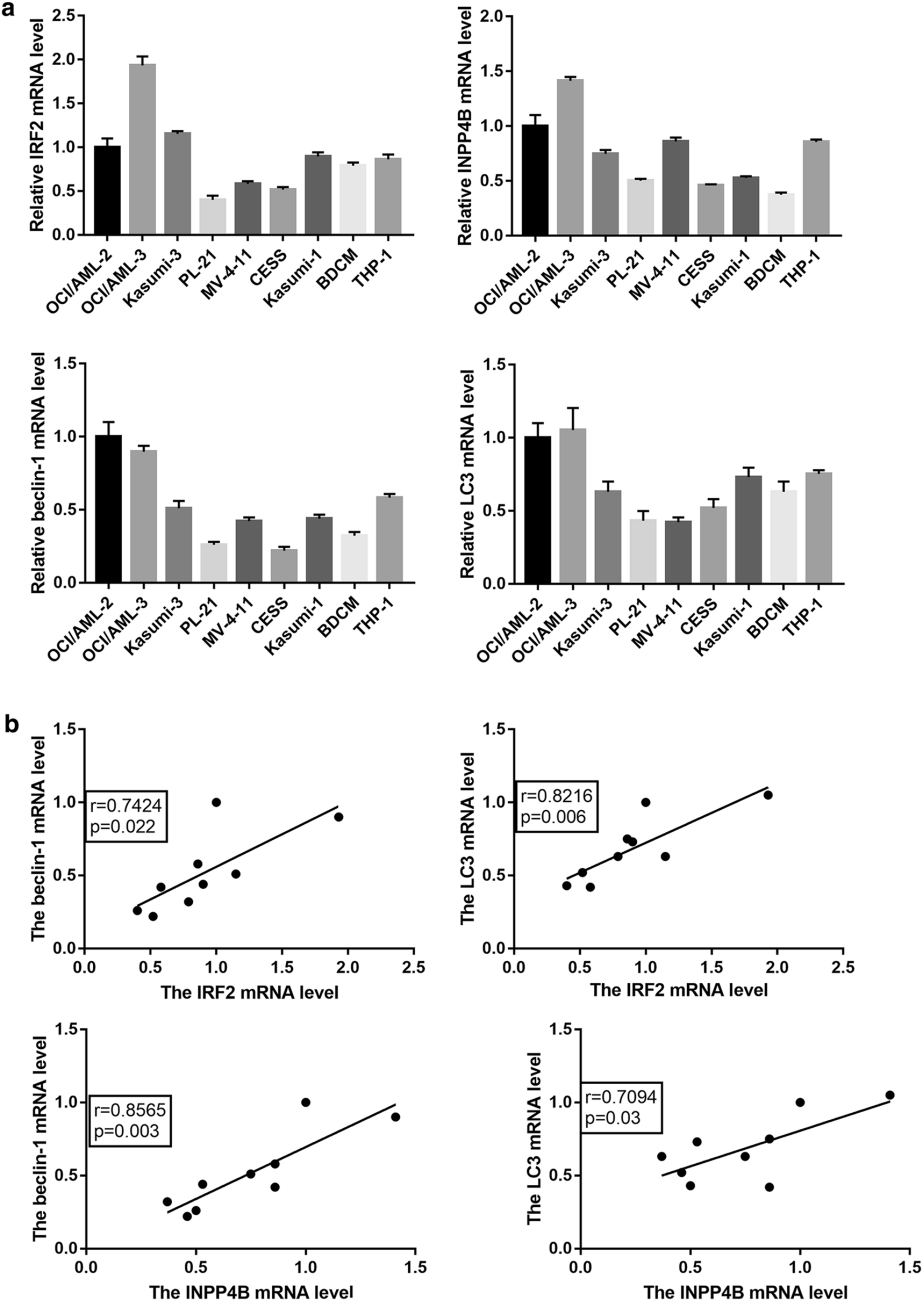
The expression and correlation of IRF2, INPP4B and autophagy-related genes in AML cell lines. The mRNA expression levels (**a**) and correlation analysis (**b**) of IRF2, INPP4B, Beclin-1 and LC3 in 9 AML cell lines OCI/AML-2, OCI/AML-3, Kasumi-3, PL-21, MV-4-11, CESS, Kasumi-1, BDCM and THP-1 cells

We firstly analyzed the mRNA expression patterns of IRF2, INPP4B, Beclin-1 and LC3 in 9 AML cell lines. The results indicated that the mRNA expression levels of IRF2 and INPP4B were similar to that of Beclin-1 and LC3 (Fig. 1a). In addition, a positive correlation was observed between the mRNA expression of IRF2 or INPP4B and Beclin-1 or LC3 (Fig. 1b). Taken together, our data suggested that IRF2 and INPP4B might be involved in the autophagy of AML cells.

### IRF2–INPP4B axis promoted autophagy in AML cells

**Fig. 2:**
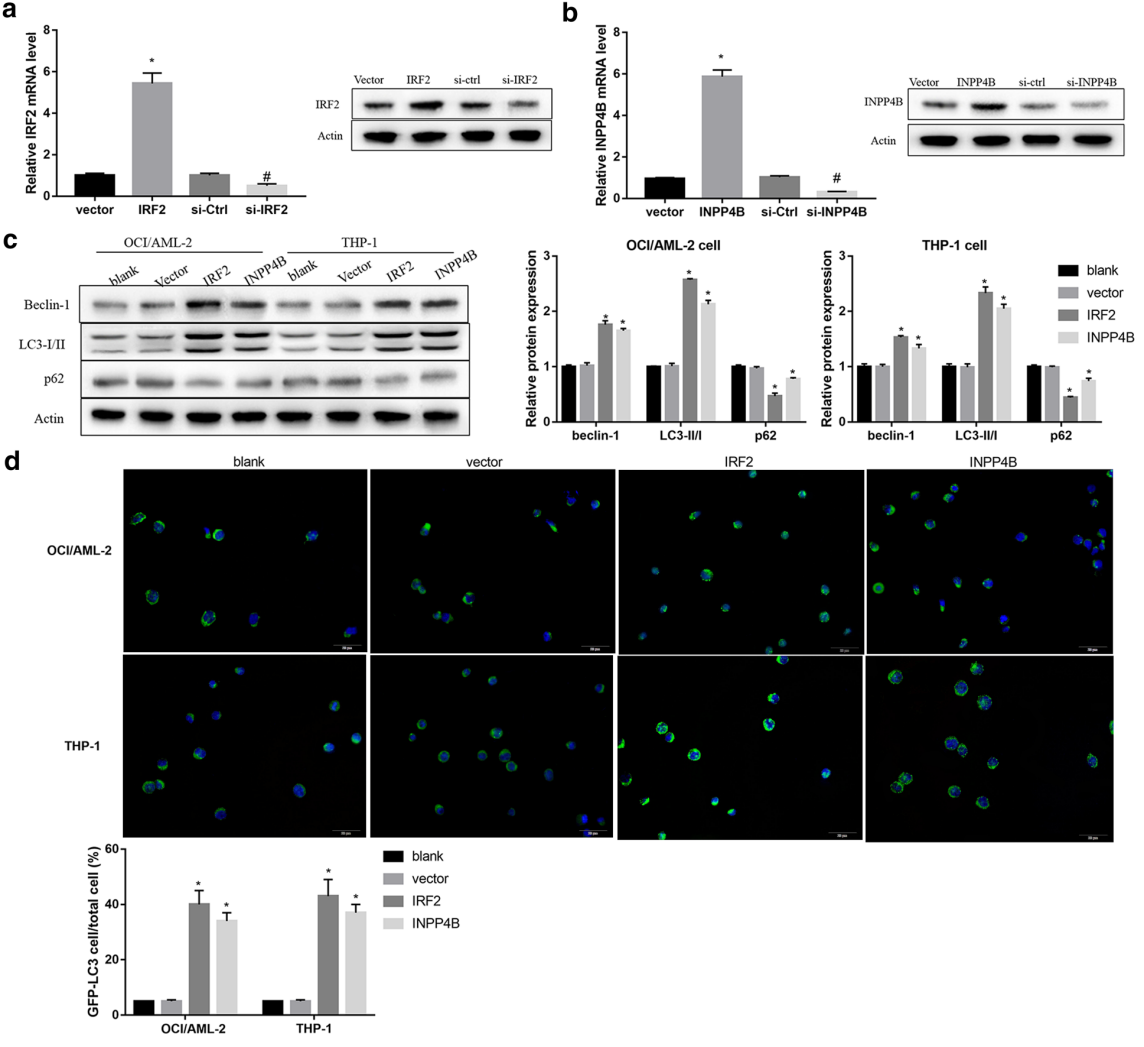
The effect of IRF2 or INPP4B overexpression on the autophagy of AML cells. **a** The mRNA and protein levels of IRF2 in OCI/AML-2 cells transfected with vector, IRF2, si-Ctrl and si-IRF2. **b** The mRNA and protein levels of INPP4B in OCI/AML-2 cells transfected with vector, INPP4B, si-Ctrl and si-INPP4B. The protein levels of Beclin-1, LC3-I, LC3-II (**c**) and P62 and the labeled number of GFP-LC3 vacuoles-positive cells (**d**) in OCI/AML-2 and THP-1 cells in the groups of blank, vector, IRF2 and INPP4B. *P < 0.05 vs. the vector-transfected cells; ^#^P < 0.05 vs. the si-Ctrl transfected cells

We next performed in vitro gain- and loss-of-function experiments in AML cells to investigate whether IRF2 and INPP4B could promote the autophagy of AML cells. Satisfactory transfection efficiency was obtained after 48 h transfection with IRF2 or INPP4B expression vector and siRNA targeting IRF2 or INPP4B plasmids as determined by qRT-PCR (Fig. 2a) and western blotting (Fig. 2b).

**Fig. 3:**
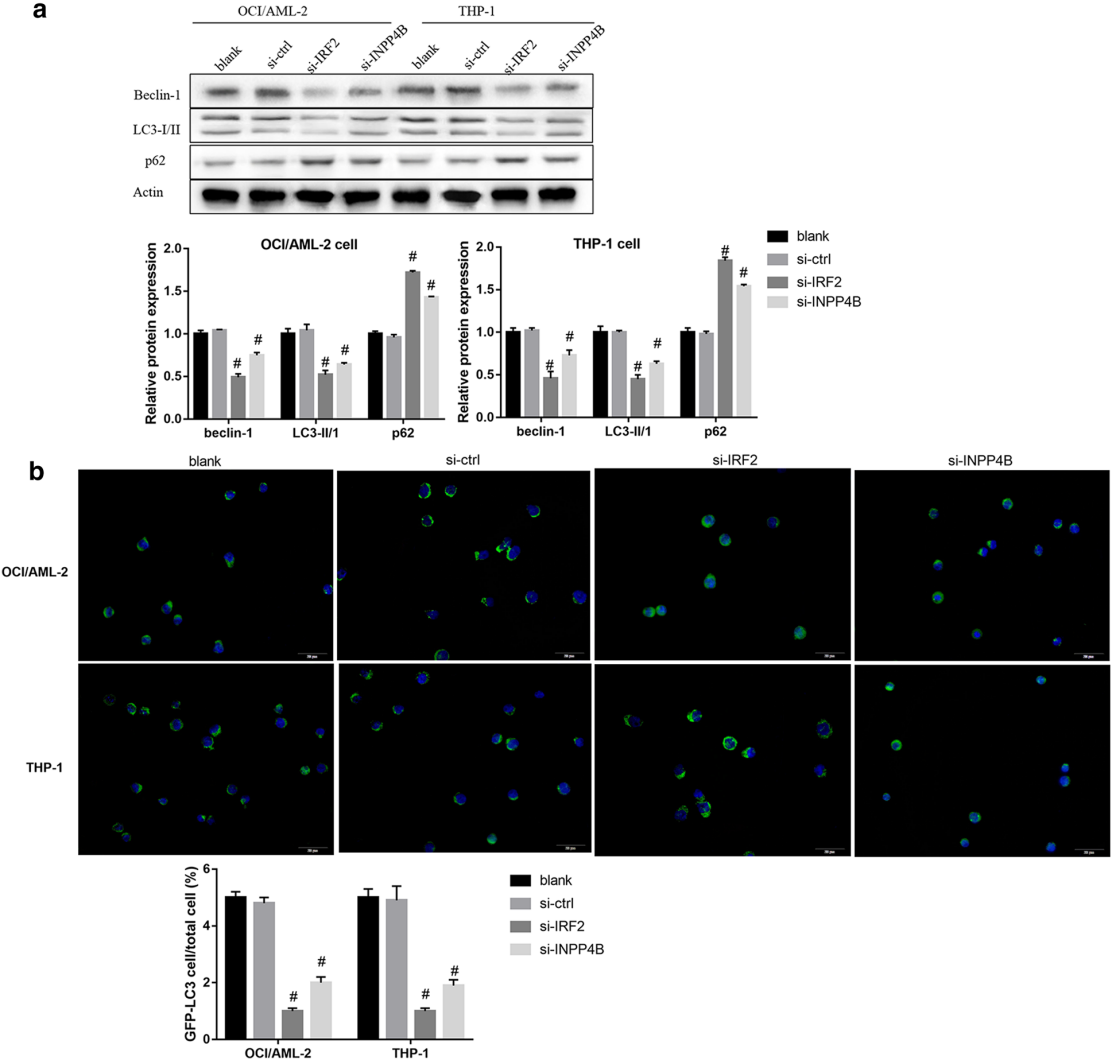
The effect of IRF2 or INPP4B knockdown on the autophagy of AML cells. The protein levels of Beclin-1, LC3-I, LC3-II and P62 (**a**) and the labeled number of GFP-LC3 vacuoles-positive cells (**b**) in OCI/AML-2 and THP-1 cells in the groups of blank, si-Ctrl, si-IRF2 and si-INPP4B. *P < 0.05 vs. the vector-transfected cells; ^#^P < 0.05 vs. the si-Ctrl transfected cells

**Fig. 4:**
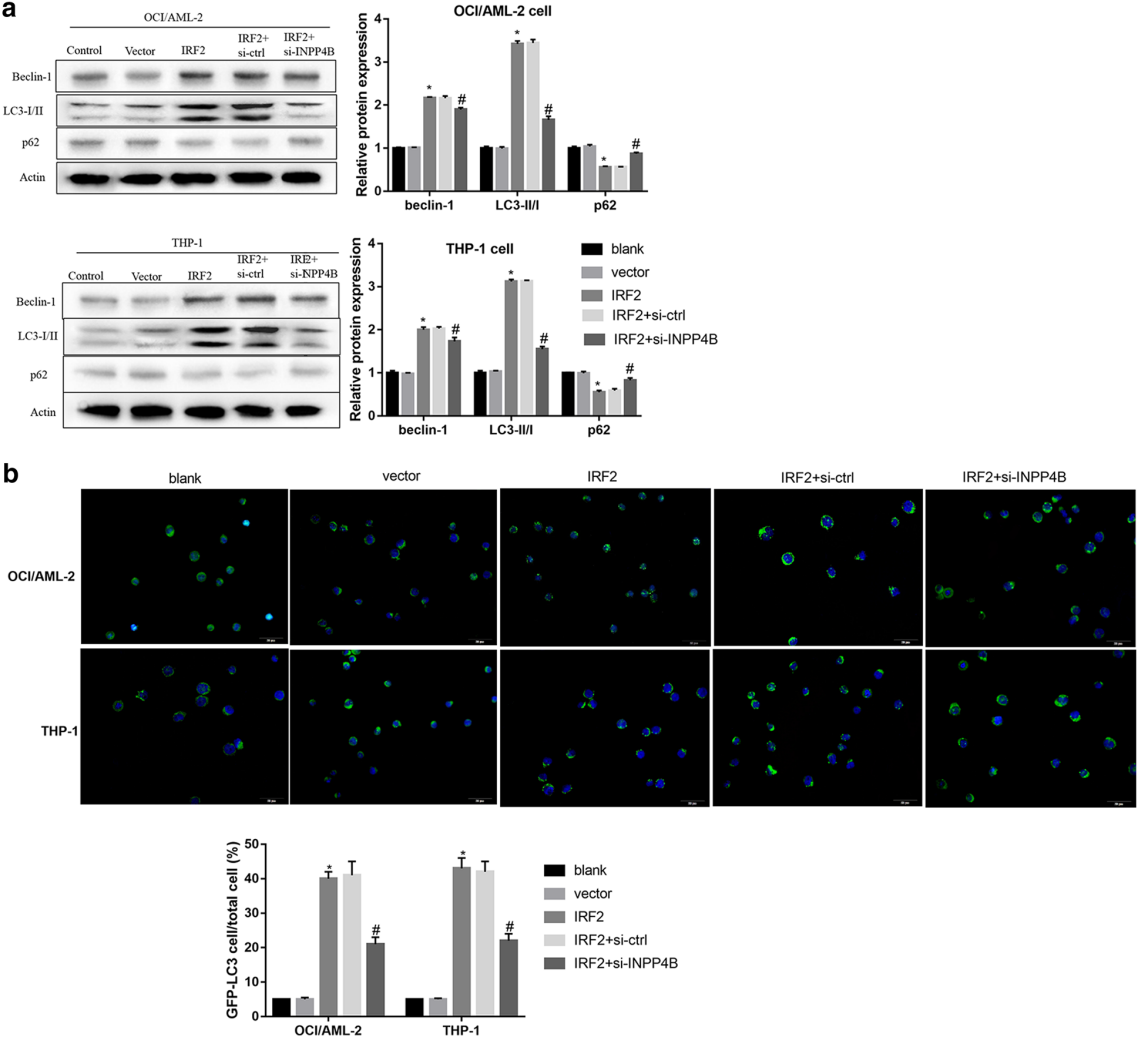
The possible mechanism of IRF2–INPP4B-induced apoptosis inhibition. The protein levels of Beclin-1, LC3-I, LC3-II and P62 (**a**) and the labeled number of GFP-LC3 vacuoles-positive cells (**b**) in OCI/AML-2 and THP-1 cells in the groups of control, vector, IRF2, IRF2 + si-Ctrl and IRF2 + si-INPP4B. *P < 0.05 vs. the vector-transfected cells; ^#^P < 0.05 vs. the IRF2 + si-Ctrl transfected cells

Furthermore, overexpression of IRF2 and INPP4B in OCI/AML-2 and THP-1 cells resulted in enhanced conversion of LC3-I into LC3-II, p62 degradation, high levels of Beclin-1 (Fig. 2c) and increased GFP-LC3-positive cells (Fig. 2d). In contrast, siRNA-mediated silencing of IRF2 and INPP4B decreased Beclin-1 protein level and LC3-II/LC3-I ratio, upregulated p62 (Fig. 3a) and reduced LC3 dots formation (Fig. 3b). Besides, INPP43 knockdown overturned the effect of IRF2 overexpression on the protein expression of autophagy-related markers (Fig. 4a) and the formation of LC3 positive puncta (Fig. 4b). Collectively, these results indicated that IRF2–INPP4B axis triggered the autophagy of AML cells.

### IRF2–INPP4B-induced autophagy inhibited apoptosis in AML cells

**Fig. 5:**
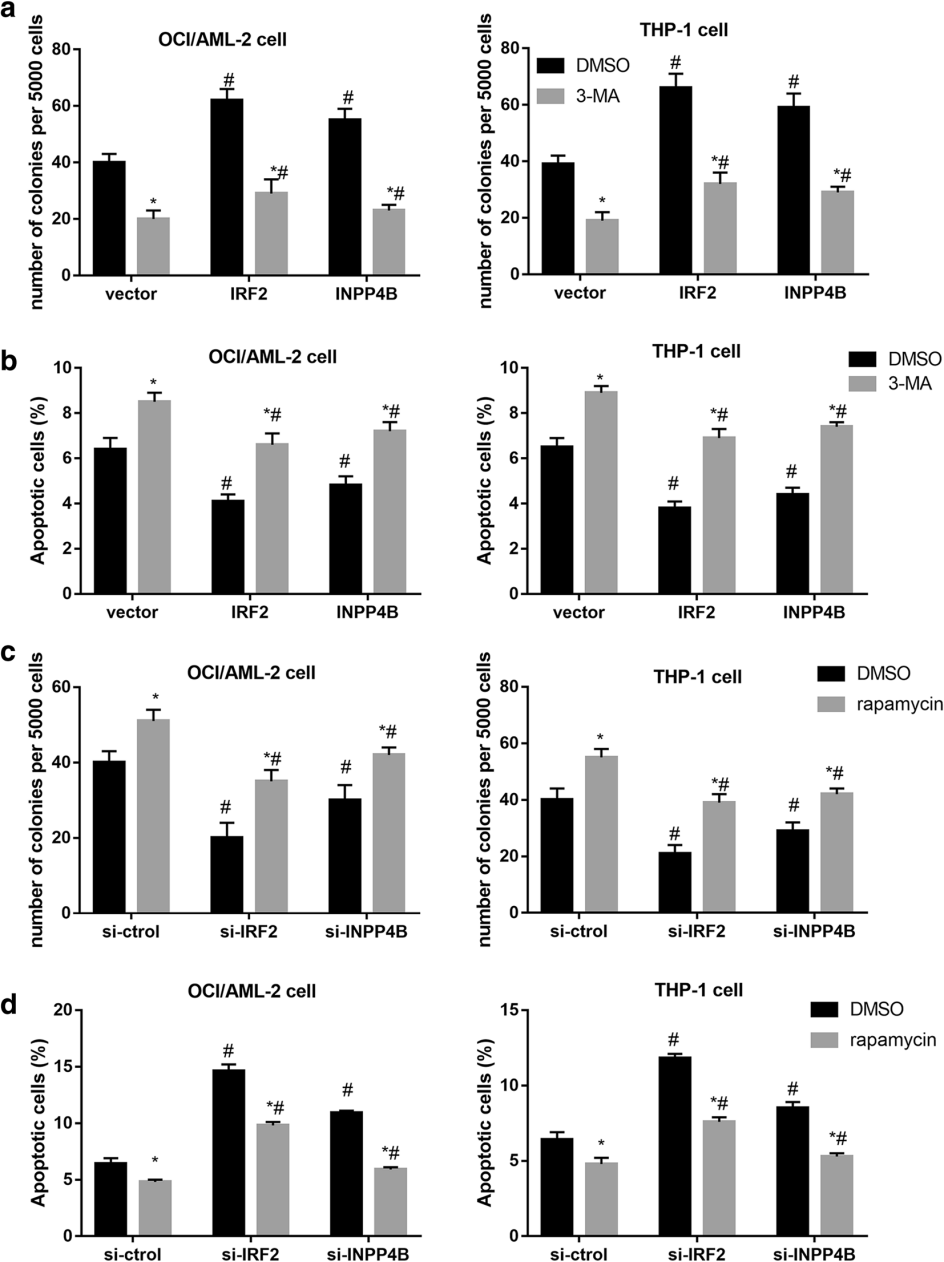
Effect of IRF2–INPP4B on proliferation and apoptosis of AML cells. The cell proliferation (**a**) and apoptosis assays (**b**) in OCI/AML-2 and THP-1 cells transfected with vector, IRF2 and INPP4B after treatment with 3-MA. The cell proliferation (**c**) and apoptosis assays (**d**) in OCI/AML-2 and THP-1 cells transfected with vector, IRF2 and INPP4B after treatment with si-Ctrl, si-IRF2 and si-INPP4B. *P < 0.05 vs. the DMSO-treatment cells; ^#^P < 0.05 vs. the si-Ctrl transfected cells

To further determine whether IRF2–INPP4B axis alleviated cell apoptosis through activation of autophagy, we used autophagy inhibitor, 3-MA to block autophagy process. Colony formation assay revealed that 3-MA suppressed and IRF2 or INPP4B overexpression promoted cell proliferation of AML cells, while enforced expression of IRF2 or INPP4B negated the inhibitory effect of 3-MA on the proliferation (Fig. 5a). Moreover, results from flow cytometry demonstrated that 3-MA promoted but IRF2 and INPP4B overexpression suppressed AML cell apoptosis (Fig. 5b). We also turned to autophagy inducer rapamycin treatment to further establish a functional importance of IRF2 and INPP4B knockdown in autophagy-related cellular phenotypes. Mechanistic analyses further revealed that rapamycin markedly facilitated the proliferation of AML cells (Fig. 5c) and suppressed apoptosis (Fig. 5d), which were reversed by IRF2 or INPP4B knockdown. Jointly, our findings manifested that IRF2–INPP4B axis inhibited apoptosis via inducing autophagy in AML cells.

## Discussion

To date, the role of autophagy in AML development has been poorly investigated. In this work, we first attempted to understand the potential mechanism that IRF2 regulated cellular apoptosis and autophagy in AML cell lines, and finally proved that IRF2 induced autophagy and inhibited apoptosis in AML cells through binding to INPP4B promoter.

In various kinds of cancers, IRF2 showed its tumor-suppressive roles, or oncogenic functions. For instance, IRF2 expression detected by immunohistochemistry was significantly downregulated in gastric cancer (GC) tissues compared to the nontumor tissues [14]. Yi et al. [15] found that hepatocellular carcinoma (HCC) patients with high IRF2 expression had lower serum alpha-fetoprotein (AFP) levels, tumor differentiation, and vascular invasion and tumor-node-metastasis (TNM) stage. On the contrary, Sakai et al. [16] demonstrated that IRF2 protein levels were observably increased in human pancreatic cancer specimens as compared with the paired normal areas of the pancreas and were associated with worse features of tumor infiltration depth and overall survival (OS). The higher expression of IRF2 were observed in colorectal cancer (CRC) tissues compared to those in paired normal tissues and were significantly associated with distant metastasis and worse OS as well as TNM stage, indicating that IRF2 functioned as an independent prognostic factor in CRC [17]. Our present study suggested that IRF2 expression was significantly upregulated in AML cell lines. More importantly, the expression of IRF2 was positively correlated with the mRNA expression of autophagy-related proteins, revealing that IRF2 might be involved in autophagy of AML cells.

Besides, IRF2 is reported to influence the occurrence and development of some cancers through proliferation, apoptosis and metastasis via altering its target genes. For example, Choo et al. [18] showed that IRF2 knockdown exhibited the obviously decreased proliferation and cell cycle and induction of polyploidy, differentiation and apoptosis in leukaemic cells. Additionally, downregulation of IRF2 significantly decreased cell proliferation of testicular embryonal carcinoma (NT2) cells by elevating p53 expression [19]. Overexpression of IRF2 can significantly inhibit the non small cell lung cancer (NSCLC) cell proliferation and invasion [20]. In addition, forced expression of IRF2 increased the expressions of proliferation-related genes cyclin D1 and proliferating cell nuclear antigen (PCNA), suggesting that IRF2 exerted oncogenic activities in human pancreatic cancer [16]. Our previous study provided the evidence that IRF2 was an important regulator of AML cell growth, colony formation and survival [13]. In accordance with previous reports, our findings revealed that IRF2 overexpression promoted cell autophagy and proliferation and inhibited cell apoptosis in AML cell lines OCI/AML-2 and THP-1, whereas IRF2 silencing led to an opposite effect, suggesting that IRF2 plays a crucial role in AML progression via autophagy induction.

Compelling evidence has delineated the carcinogenesis of INPP4B in breast cancer [21], laryngeal cancer [22] and melanoma [23]. Recently, accumulating evidence has strongly implied that INPP4B served as independent prognostic marker and were associated with colony formation, proliferation and chemotherapy resistance in AML patients [24–26]. The previous research has elucidated that IRF2 elevated the activity of INPP4B promoter by directly binding to its promoter to increase INPP4B expression in AML cells [13]. There was a positive correlation between INPP4B and Beclin-1 as well as LC3 mRNA expressions in AML cell lines. Besides, the overexpression of INPP4B promoted cell autophagy and proliferation and reduced cell apoptosis in AML cells, while lower expression level of INPP4B by si-INPP4B significantly promoted cell apoptosis and suppressed autophagy. Ko et al. found that autophagy inducer rapamycin restricted a feedback loop of NLRP3 inflammasome-p38 MAPK-NF-κB pathways in autophagy- and p62-dependent manners [27]. In addition, previous studies uncovered that rapamycin, as a mTOR kinase inhibitor involved multiple signaling pathways such as Ras/MEK/ERK, MAPK, JAK/STAT and Notch-1 pathways [28, 29]. Thus, in the present study, the silencing of IRF2 or INPP4B could partially but not completely reverse the apoptosis-promoting effect of rapamycin.

## Conclusions

In summary, we provided the first demonstration that IRF2–INPP4B axis inhibited the apoptosis of AML cells via inducing autophagy in vitro, and thus may be a new target for gene therapy in AML.

## Abbreviations

3-MA: 3-methyladenine
AFP: alpha-fetoprotein
AML: acute myeloid leukemia
Atg5: autophagy-related gene 5
CRC: colorectal cancer
FBS: fetal bovine serum
GC: gastric cancer
GFP: green fluorescent protein
HCC: hepatocellular carcinoma
IRF: interferon regulatory factor
IRF2: interferon-regulatory factor 2
INPP4B: inositol polyphosphate-4-phosphatase, type-II
LC3: light chain 3
NSCLC: non small cell lung cancer
OS: overall survival
PCNA: proliferating cell nuclear antigen
QRT-PCR: quantitative real time PCR
siRNA: small interference RNA
TNM: tumor-node-metastasis
